# Reflectance Confocal Microscopy in the Diagnosis of Onychomycosis: A Systematic Review

**DOI:** 10.3390/jof8121272

**Published:** 2022-12-02

**Authors:** Sophie Soyeon Lim, Bo Ri Kim, Je-Ho Mun

**Affiliations:** 1 Alfred Health, Melbourne 3004, Australia; 2 Department of Dermatology, Seoul National University College of Medicine, Seoul 03080, Republic of Korea; 3 Department of Dermatology, Seoul National University Bundang Hospital, Seongnam 13620, Republic of Korea; 4 Department of Dermatology, Seoul National University Hospital, Seoul 03080, Republic of Korea

**Keywords:** diagnosis, nail diseases, microscopy, confocal, fungi, onychomycosis, reflectance confocal microscopy

## Abstract

Accurately diagnosing onychomycosis is vital, as therapy is time-consuming and accompanied by multiple adverse effects. Reflectance confocal microscopy (RCM), in contrast to traditional mycological testing, is a noninvasive, point-of-care tool that can rapidly identify fungal lesions. This systematic review aims to understand the utility of RCM in evaluating onychomycosis and follows the Preferred Reporting Items for Systematic Reviews and Meta-Analyses guidelines. A systematic search of four databases was conducted. A total of five articles—three prospective cohort studies and two case reports—which reported RCM findings in nails clinically suspicious for onychomycosis were analyzed. Fungal hyphae or spores were visualized on RCM in 67 (81.7%) of the 82 mycologically confirmed cases of onychomycosis. Terms used to describe hyphae included bright, linear, lengthy, thready-like, branching and filamentous. Spores were described as bright, roundish structures with high reflection. The three cohort studies demonstrated RCM had a sensitivity of 52.9–91.7, a specificity of 57.58–90.2%, a positive predictive value of 61.1–88.6% and a negative predictive value of 68.0–90.5%. In conclusion, existing studies demonstrate how RCM can assist the diagnosis of onychomycosis at the bedside. Larger studies incorporating multiple testing modalities to confirm the diagnosis of onychomycosis are warranted to further explore the diagnostic utility of RCM.

## 1. Introduction

Onychomycosis requires a prompt diagnosis, as treatment delays can cause nail dystrophy and nail loss [1]. As the treatment for onychomycosis has various adverse effects, including gastrointestinal disturbances, drug interactions and hepatotoxicity, accurately diagnosing onychomycosis is crucial to prevent patients without onychomycosis from being prescribed such therapies. Therefore, detecting fungi in the nail plate is essential to confirm the diagnosis before initiating treatment. Reflectance confocal microscopy (RCM) is a noninvasive imaging tool that allows a real-time examination of the skin down to the papillary dermis. It can help diagnose malignant skin tumors [2,3] as well as inflammatory and infectious dermatoses [4]. Notably, clinicians have applied RCM to evaluate nails for onychomycosis. Unlike dermoscopy, RCM can visualize fungal elements at the bedside and thus has the potential to noninvasively diagnose onychomycosis. Moreover, as RCM is a point-of-care examination tool, it can diagnose onychomycosis faster than existing mycological tests. The objective of this systematic review was to evaluate the current evidence on the use of RCM in diagnosing onychomycosis.

## 2. Materials and Methods

This study followed the Preferred Reporting Items for Systematic Reviews and Meta-Analyses (PRISMA) guidelines [5]. Articles published in PubMed, Embase, Scopus and Cochrane databases from inception to 6 May 2022 were screened. The search terms included onychomycosis (OR its related keywords) AND confocal microscopy (OR its related keywords) (Table 1). No filters or limits were used. The articles were independently reviewed by two authors (SL and BRK). The authors of the reviewed literature were not contacted. Review articles, opinion articles, guidelines and consensus documents were excluded. Articles such as that of Vanstone et al. [6] that incorporated RCM into their methods to investigate a research question not directly related to the RCM features of onychomycosis were excluded. A quality assessment was conducted using the Quality Assessment of Diagnostic Accuracy Studies 2 (QUADAS-2). Data collected from the articles were the name of the first author, publication date, study type, number and characteristics of included cases, methods, type of RCM used and study results. The study protocol was not registered.

## 3. Results

**Figure 1:**
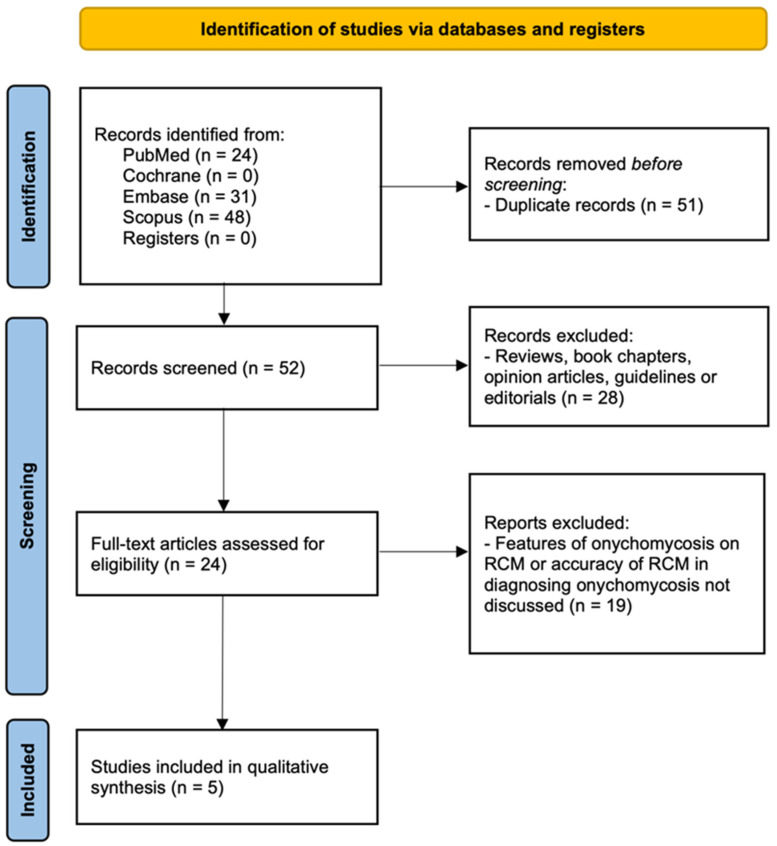
PRISMA Diagram on Search Strategy.

**Figure 2:**
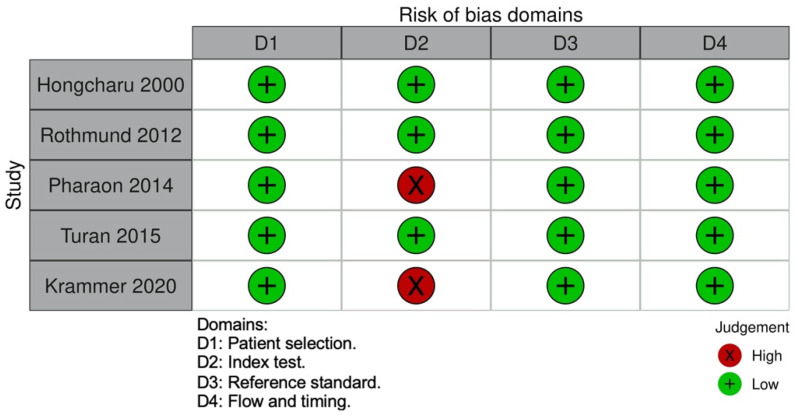
QUADAS-2 Risk of Bias Assessment.

A total of 103 articles were found across the four databases, of which 51 were duplicates (Figure 1). Twenty-four studies were full-text articles, and five of them reported RCM features of onychomycosis (Table 2) [7,8,9,10,11]. The risk of bias in the index test in two articles [9,11] was deemed high; Pharaon et al. used two different RCM devices and Krammer et al. used an acridine orange dye and a fluorescence filter to examine nail specimens. The risk of bias across the other domains of the included articles was deemed to be low (Figure 2).

Two articles [8,9] utilized only in vivo RCM, two articles [7,10] used both in vivo and in vitro RCM and one article [11] only utilized ex vivo RCM. Two articles [7,10] were case reports of mycologically confirmed cases of onychomycosis, and three [8,9,11] were prospective cohort studies that compared RCM to existing mycological tests (potassium hydroxide (KOH) testing, fungal culture, histology or PCR). Rothmund et al. also compared RCM to optical coherence tomography [8]. The method of diagnosing onychomycosis varied amongst the studies. Pharaon et al. and Turan et al. diagnosed onychomycosis in clinically suspicious nails which were KOH and culture-positive [9,10]. Hongcharu et al. diagnosed onychomycosis when a nail clinically suspicious for onychomycosis was KOH positive [7], and Krammer et al. diagnosed onychomycosis on clinical examination and a positive result on either KOH, culture or histology [11]. Finally, Rothmund et al. diagnosed onychomycosis based on mycological tests only; they diagnosed onychomycosis if a nail was positive on either PCR, culture or histology [8].

All articles described the visualization of hyphae on RCM. Hyphae were described to be bright and linear, lengthy, threadylike, branching or filamentous. Bright roundish, sporelike structures with a high reflection were also described in two articles [8,9]. RCM was able to identify hyphae or spores in 67 (81.7%) of the 82 mycologically confirmed cases of onychomycosis. According to the three cohort studies [8,9,11], RCM has a sensitivity of 52.9–91.67%, specificity of 57.58–90.2%, positive predictive value of 61.11–88.6% and negative predictive value of 68.0–90.48% (Table 2). Pharaon et al. reported that the handheld imager was more sensitive (60%) than the standard RCM device (50%), but this difference was not statistically significant [9].

## 4. Discussion

Detecting fungi in nails is the key to diagnosing onychomycosis [1,12]. Oral antifungal agents are the mainstay of onychomycosis treatment; however, they have multiple disadvantages, including an extended treatment duration lasting at least 2–3 months, drug interactions, gastrointestinal disturbances, and hepatotoxicity [13]. Although topical antifungal agents do not have such disadvantages, they have limited efficacy. They are generally prescribed in early or superficial cases of onychomycosis [13]. Coupling topical antifungals with laser therapy improves efficacy; however, further research is required before integrating this emerging form of treatment into clinical practice [14]. Combination therapy with oral medication, topical medications, and devices can be used to enhance efficacy and safety. However, a recent systematic review did not support it as a first-line treatment option [15]. Considering these facts, accurately diagnosing that the patients have onychomycosis before commencing treatment is critical.

Our systematic review demonstrated how RCM detected fungal hyphae or spores in 81.7% of mycologically confirmed cases of onychomycosis. Existing studies demonstrated that the specificity of ex vivo RCM (57.6%) may be lower than that of in vivo RCM (81.0% or 90.2%). However, ex vivo RCM was reported to be more sensitive (91.7%) than in vivo RCM (52.9% or 79.5%). The fact that ex vivo RCM improves sensitivity but reduces specificity may partly be due to the method used for tissue preparation and specimen examination. Nail specimens were stained with acridine orange, and a digital staining mode on the RCM, which mimics hematoxylin and eosin staining commonly used prior to histological assessment, was incorporated [11]. This may have improved the detection of fungal hyphae on RCM, increasing the true-positive rate.

Among the in vivo RCM studies, the sensitivity reported by Pharaon et al. (53%) was significantly lower than that reported by Rothmund et al. (79%) [8,9]. This could be due to the use of two different RCM devices in these studies, with the Vivascope 3000 handheld imager achieving a higher sensitivity (60%) than the Vivascope 1500 (50%) [9]. Rothmund et al. also utilized the Vivascope 1500 to evaluate all their cases of onychomycosis, using the same 830 nm laser in reflection mode and a 500 × 500 μm single image sections [8].

Our study was limited by the small number of studies that could be included. Further studies with larger sample sizes are needed to improve our understanding of the diagnostic utility of RCM.

## 5. Conclusions

RCM is a noninvasive point-of-care examination tool that allows clinicians to visualize fungal hyphae in the nails before initiating onychomycosis treatment. The study highlights how RCM can aid the evaluation of onychomycosis at the bedside. Further studies are warranted to integrate RCM into clinical practice for diagnosing onychomycosis.

## Figures and Tables

**Table 1:** Search strategy utilized for each database.

Database	Search Strategy
PubMed	Onychomycosis AND confocal microscopy
Embase	(Onychomycosis/OR fingernail onychomycosis/OR toenail onychomycosis/OR onychomycosis.mp) AND (reflectance confocal microscopy/OR confocal microscopy/OR reflectance confocal microscopy.mp OR confocal microscopy.mp)
Scopus	TITLE-ABS-KEY (onychomycosis) AND TITLE-ABS-KEY (confocal AND microscopy)
Cochrane	Onychomycosis in Title Abstract Keyword AND confocal microscopy in Title Abstract Keyword—(Word variations have been searched)

**Table 2:** Characteristics of studies that investigated reflectance confocal microscopy in diagnosing onychomycosis.

First Author and Publication Year	Study Type	Number of Cases	Methods	Type of RCM Used	RCM Findings	Number of Cases with Positive RCM Findings	Diagnostic Accuracy
Hongcharu, 2000 [7]	Case report	1 case of onychomycosis diagnosed on clinical assessment and KOH testing	In vivo and in vitro confocal infrared microscopy	RCM assembled using a range of laser-producing, scanning and detecting devices. A 1064 nm Nd:YAG laser was used.	- Network of branched hyphae in both in vivo and in vitro confocal images	1 (100%)	ND
Rothmund, 2012 [8]	Prospective cohort	50 clinically suspicious cases of onychomycosis and 10 cases of controls (patients with nail disorders that did not display the typical clinical features of onychomycosis)	Comparison of the diagnostic accuracy of KOH, fungal culture, PCR, histology, in vivo RCM, and optical coherence tomography Onychomycosis diagnosed after positive KOH, culture, PCR or histology.	VivaScope 1500 with 830 nm laser in reflection mode	- Hyphae: lengthy, threadylike structures with high reflection - Sporelike aggregates with high reflection	35 (58.3%): 34 in nails suspicious for onychomycosis; 1 in control group	Sensitivity 79.5% Specificity 81.0% PPV 88.6% NPV 68.0%
Pharaon, 2014 [9]	Prospective cohort	58 clinically suspicious cases of onychomycosis	Comparison of the diagnostic accuracy of KOH, fungal culture, and in vivo RCM. Two different RCM devices were used: a standard device and a handheld imager. Onychomycosis diagnosed after positive KOH and culture.	VivaScope 1500 and VivaScope 3000 (Lucid Inc, New York): both using an 830 nm laser in reflection mode	- Septate hyphae: aggregates of bright branching filamentous structures - Arthroconidia: bright roundish structures	13 (22.4%)	Sensitivity 52.9% Specificity 90.2% PPV 69.2% NPV 82.2%
Turan, 2015 [10]	Case report	1 case of onychomycosis diagnosed on KOH testing and fungal culture	In vivo and in vitro confocal laser scanning microscopic examination.	Multilaser RCM using single wavelength laser of 786 nm	- Branching hyphae visualized as refractile, bright, linear structures situated just below the nail plate surface	1 (100%)	ND
Krammer, 2020 [11]	Prospective cohort	57 clinically suspicious cases of onychomycosis	Comparison of the diagnostic accuracy of KOH, fungal culture, histology, and ex vivo RCM Onychomycosis diagnosed after positive KOH, culture or histology	Prototype VivaScope 2500 M-G4 (Lucid Inc, New York): using 488 nm (blue) and 785 nm (red) lasers	Hyphae demonstrated in images (specific describing words were not used)	36 (63.2%)	Sensitivity 91.7% Specificity 57.6% PPV 61.1% NPV 90.5%

ND = not done; PPV = positive predictive value; NPV = negative predictive value.

## Data Availability

All data analyzed during this study are included in this article. Further inquiries can be directed to the corresponding author.
